# Metabolic Syndrome Clusters and Arterial Stiffness: Unraveling Early Predictors of Cardiovascular Risk in a Follow-Up Study

**DOI:** 10.3390/jcdd12090332

**Published:** 2025-08-29

**Authors:** Agnė Jucevičienė, Ligita Ryliškytė, Jolita Badarienė, Aleksandras Laucevičius

**Affiliations:** 1 Clinic of Cardiovascular Diseases, Institute of Clinical Medicine, Faculty of Medicine, Vilnius University, M. K. Čiurlionio Str. 21, LT-03101 Vilnius, Lithuania; 2 Vilnius University Hospital Santaros Klinikos, Santariškių Str. 2, LT-08406 Vilnius, Lithuania; 3 State Research Institute Centre for Innovative Medicine, Santariškių Str. 5, LT-08406 Vilnius, Lithuania

**Keywords:** pulse wave velocity, aortic stiffness, cardiovascular events, myocardial infarction, stroke, cardiovascular death, metabolic syndrome, metabolic syndrome clusters

## Abstract

Background: The aim of this study was to assess the association between different metabolic syndrome (MetS) component clusters, arterial stiffness as measured by aortic pulse wave velocity (aPWV) and cardio-ankle vascular index (CAVI), and the incidence of major cardiovascular events during long-term follow-up. Methods: The prospective cohort study included 5307 participants with MetS, aged 40 to 64 years, who had no evident cardiovascular disease and were enrolled in the Lithuanian High Cardiovascular Risk primary prevention program. All participants were followed up for an average of 4.57 ± 2.74 years to monitor the occurrence of major cardiovascular events. Arterial stiffness was assessed using aPWV and CAVI measurements. The associations between different MetS component clusters, arterial stiffness, and cardiovascular outcomes were analyzed. Results: During the follow-up period, 3.34% of the subjects experienced a major cardiovascular event. Individuals meeting four MetS criteria had a higher risk of events compared to those meeting three. Elevated triglycerides and elevated glucose were each significantly associated with increased risk. Specific MetS combinations, particularly clusters involving WTHB (increased waist circumference [W], elevated triglycerides [T], decreased HDL cholesterol [H], and elevated blood pressure [B]), as well as WBG (waist circumference, blood pressure, and glucose [G]), were significantly associated with cardiovascular events. The cross-sectional analysis also revealed that arterial stiffness, assessed as aPWV, was significantly higher in subjects with the WBG, WTBG, and WTHBG clusters. Meanwhile, higher CAVI was associated with the WTBG cluster. In the logistic regression analysis, the presence of the following clusters was linked to more than twice increased odds for having extremely stiff arteries: WTBG (OR = 2.351) and WTHBG (OR = 2.201) for aPWV values above the 95th percentile (>11.3 m/s) and WTB (OR = 2.096) for CAVI values above the 95th percentile (>10.2). Conclusions: Our findings demonstrate that higher risk of CV events is associated with increased arterial stiffness and higher number of MetS components present, as well as with the presence of specific MetS components; in particular, increased levels of triglycerides and glucose. Furthermore, the cross-sectional analysis demonstrated that subjects with the unfavorable combination of MetS components, such as WTBG, WTHBG, and WTB, are more than twice as likely to have extremely stiff arteries.

## 1. Introduction

Metabolic syndrome (MetS) emerges as a crucial factor driving the ongoing global cardiovascular crisis, significantly heightening the susceptibility to type 2 diabetes [1,2], cardiovascular disease [3], and premature mortality [4]. A comprehensive meta-analysis conducted in 2022, encompassing a substantial cohort of 28,193,768 participants, unveiled a notable variability in the global prevalence of MetS, ranging from 12.5% (95% CI: 10.2–15.0) to 31.4% (95% CI: 29.8–33.0), depending on the applied definition. Notably, the prevalence exhibited a marked elevation in the Eastern Mediterranean Region and the Americas, demonstrating a direct correlation with the economic status of the countries [1].

Numerous investigations have indicated that individuals diagnosed with MetS often display heightened arterial stiffness levels, which contribute to the emergence and advancement of cardiovascular complications such as hypertension, atherosclerosis, and coronary artery disease [5,6]. Arterial stiffness serves as more than just a reflection of structural alterations within the arterial wall but also as a marker of endothelial dysfunction and impaired vascular smooth muscle function, both of which play pivotal roles in the pathogenesis of cardiovascular disease (CVD) [7,8].

Moreover, increased arterial stiffness functions as a predictor of cardiovascular events and mortality, underscoring its clinical relevance in the risk assessment and treatment among individuals afflicted with MetS [9]. The evaluation of arterial stiffness holds promise in offering crucial insights into the early identification and surveillance of cardiovascular risk among MetS patients, facilitating timely interventions to forestall the onset or progression of CVD.

A study conducted by Scuteri et al. examined the variability in the manifestation of metabolic syndrome across different European populations. Through the analysis of diverse cohorts, the research identified distinct clusters of MetS components, emphasizing regional differences in the prevalence of risk factors [10]. Another large-scale cross-sectional study involving 20,570 individuals from nine cohorts (eight from various European countries and one from the United States) explored the association between metabolic syndrome and arterial stiffness across multiple populations. By utilizing data from multiple countries, the study assessed how MetS components contribute to increased arterial stiffness, a well-established predictor of cardiovascular events. Performed assessment revealed strong relationship between increased arterial stiffness and three different combinations of MetS components: WTB (increased waist circumference, elevated triglycerides, and high blood pressure), WBG (increased waist circumference, high blood pressure, and elevated glucose level), WTBG (increased waist circumference, elevated triglycerides, high blood pressure, and elevated glucose level).

This transnational analysis provides valuable insights into the role of MetS for affecting the surrogate markers of cardiovascular risk, emphasizing the importance of early detection and management of its components to reduce arterial stiffness [11]. However, only a longitudinal study can verify the association between the “risky” clusters of MetS components and increased rate of CV events.

Rodriguez-Colon et al. demonstrated that distinct clusters of metabolic syndrome components exert differential effects on certain health outcomes. In their analysis, individuals presenting with both high blood pressure and elevated fasting glucose faced the highest risk for incident stroke relative to other component combinations [12,13]. Despite that, there is a notable gap in the literature regarding the relationship between metabolic syndrome component clusters and a heightened risk of other cardiovascular events, such as myocardial infarction and sudden cardiac death.

The present study aims to make up for this lacuna by comparing the associations between various combinations of metabolic syndrome components, arterial markers, and major cardiovascular events, including myocardial infarction, stroke, and cardiovascular mortality. Recognizing and analyzing these clusters is essential for advancing personalized medical interventions. By identifying specific clusters, healthcare providers can design targeted prevention and treatment strategies tailored to the unique risk profiles of individuals with metabolic syndrome.

## 2. Materials and Methods

### 2.1. Subjects and Study Design

A total of 5307 patients diagnosed with metabolic syndrome were included in our prospective study. These patients were exclusively recruited from a single specialized healthcare institution, Vilnius University Hospital Santaros Klinikos, within the scope of the LitHiR program [14]. According to the LitHir protocol, these subjects were referred to the Vilnius University Santaros Klinikos from the primary care centers.

Eligibility for study participation was determined based on the following criteria:

### 2.2. Inclusion Criteria

**Age:** women aged 50–65 years and men aged 40–55 years.

**Medical History:** no prior history of cardiovascular disease.

**Presence of MetS**.

### 2.3. Exclusion Criteria

**Coronary Heart Disease (CHD):** history of myocardial infarction (MI), unstable angina, exertional angina with positive stress test results, coronary pathology detected through coronary angiography or multi-slice computed tomography angiography, coronary artery bypass grafting or angioplasty, and acute coronary events.

**Cerebrovascular Disease:** history of acute cerebrovascular events or confirmed stenosis in the carotid arteries.

**Peripheral Artery Disease:** history of acute ischemic syndrome, verified chronic limb ischemia, or aortic aneurysm.

**End-stage disease:** end-stage oncological condition or any other terminal somatic ailment.

### 2.4. Clinical Assessment

Blood pressure was measured, using an oscillometric semiautomatic device (Schil-ler Argus VCM) with a standard bladder (12–13 cm long and 35 cm wide), and validated with a standardized mercury sphygmomanometer. Blood pressure was measured twice on the right arm after 5 min of rest, with the higher value being recorded. Initial assessments confirmed no significant inter-arm differences. Waist circumference was measured in centimeters at the midpoint between the lowest rib and iliac crest, using a non-stretchable tape parallel to the floor at the end of a gentle exhalation.

Additionally, a venous blood sample was collected after a 12 h fast for the evaluation of total cholesterol, low-density lipoprotein cholesterol, high-density lipo-protein cholesterol, triglyceride, and glucose levels.

### 2.5. Metabolic Syndrome Diagnosis Criteria

The diagnosis of metabolic syndrome was established using the updated National Cholesterol Education Program Adult Treatment Panel III (NCEP ATPIII) criteria [15], requiring the presence of at least three of the following:

**Waist circumference:** ≥102 cm for men, and ≥88 cm for women.

Triglyceride level: ≥1.7 mmol/L.

**High-density lipoprotein cholesterol level:** <1.03 mmol/L for men, and <1.29 mmol/L for women.

**Blood pressure:** ≥130/85 mmHg or the use of antihypertensive medication for an individual with a history of arterial hypertension.

**Fasting plasma glucose level:** ≥5.6 mmol/L.

### 2.6. Ethical Statement

The study was conducted in accordance with the principles of the Declaration of Helsinki, received approval from the Regional Ethics Committee (Permission No. 2019/3-1104-603), and the need to obtain informed patient consent was waived.

### 2.7. The Assessment of Arterial Markers

Non-invasive methods were employed to evaluate arterial markers suggestive of subclinical atherosclerosis among all subjects. This included assessing arterial stiffness and wave reflection parameters. Patients were instructed to abstain from smoking and physical activity for at least 2 h before the examination. Vascular assessment commenced following a 10 min rest period in a supine position.

The evaluation of arterial stiffness and wave reflection parameters involved conducting the following tests.

An applanation tonometry system (SphygmoCor, AtCor Medical, version 8.0, Sydney, Australia) in conjunction with a high-fidelity micromanometer (Millar Instruments, Houston, TX, USA), was utilized to evaluate pulse wave velocity (PWV) and wave reflection. The system was positioned on the skin surface overlying the carotid and femoral arteries’ projection to capture pulse pressure waveforms. In addition, the direct distance between the common carotid artery and the femoral artery was determined, and brachial blood pressure was recorded. This data, combined with the electrocardiogram, facilitated the computation of the primary arterial stiffness parameter, i.e., the aortic (carotid–femoral) pulse-wave velocity (aPWV) [16,17].

Measurement of the cardio-ankle vascular index (CAVI) was performed utilizing the VaSera VS-1000 (Fukuda Denshi Co. Ltd., Tokyo, Japan). Following a 5 min supine stabilization period, blood pressure readings were obtained from the posterior tibial and brachial arteries. Evaluation of the CAVI involved the placement of four blood pressure cuffs on the four extremities. Electrocardiography electrodes were affixed to both arms, while a microphone was situated on the sternum in the second intercostal space. Following a 10 min supine stabilization period, electrocardiography and phonocardiography were monitored, and CAVI was determined using Bramwell–Hill’s equation [18]: $CAVI=a\times \frac{2\rho }{\Delta P}\times \ln\left(\frac{Ps}{Pd}\right)\times {PWV}^{2}+b$. In the provided equation, ‘Ps’ denotes systolic blood pressure; ‘Pd’ denotes diastolic blood pressure; ‘ΔP’ denotes the difference between systolic and diastolic blood pressure; ‘PWV’ denotes the cardio-ankle pulse wave velocity; ‘ρ’ denotes blood viscosity; and ‘a’ and ‘b’ are constants utilized for converting the CAVI value to a value obtained using the Hasegawa method.

### 2.8. MetS Component Combinations

All metabolic syndrome components have been assigned a single letter based on the criteria used:

**W—Waist circumference:** ≥102 cm for men, and ≥88 cm for women.

**T—Triglyceride level:** ≥1.7 mmol/L.

**H—High-density lipoprotein cholesterol level:** <1.03 mmol/L for men, and <1.29 mmol/L for women.

**B—Blood pressure:** ≥130/85 mmHg or the use of antihypertensive medication for an individual with a history of arterial hypertension.

**G—Fasting plasma glucose level:** ≥5.6 mmol/L.

When assessing the influence of different metabolic syndrome component combinations on the risk of experiencing a major cardiovascular event, a table (Table 1) of possible combinations was created and encoded based on the components present when diagnosing MetS. Metabolic component combinations with prevalence lower than 5% of population were combined into a separate group when comparing different clusters.

### 2.9. Follow-Up and Clinical Outcomes

Patients were monitored for cardiovascular events over an average period of 4.57 ± 2.74 years. The Lithuanian National Death Registry and the National Healthcare Fund Disease and Services Database were used to retrieve the outcome data. The assessment of outcomes included myocardial infarction, stroke, and CV death (comprising both ischemic and hemorrhagic strokes) as CV events.

### 2.10. Statistical Analysis

The statistical analysis was carried out utilizing the R statistical computing environment (version 4.4.3; The R Foundation for Statistical Computing 2025).

Relationships between MetS components, MetS clusters, and major cardiovascular events were assessed using Fisher’s exact test.

To compare means between subgroups of subjects, ANOVA analysis followed by Bonferroni test was used. Least square means (±standard error) were calculated with the use of ANCOVA analysis to compare aPWV and CAVI values between various clusters of metabolic syndrome components.

Multivariable logistic regression analysis was adopted to identify which combinations of metabolic syndrome components were significant determinants of extremely stiff arteries. Additional models were run in men and women separately.

ANCOVA and logistic regression analyses included adjustment for age, gender, non-HDL cholesterol, smoking, and diabetes due to earlier established associations between these variables and cardiovascular events in our previous study [19].

All *p*-values were calculated as 2-tailed, with the level of significance set at 0.05.

## 3. Results

### 3.1. Baseline Characteristics

The mean age of the included participants was 52.9 ± 6.46 years, with 42.2% (2241) being men and 57.8% (3066) being women. All participants were monitored for cardiovascular events for an average duration of 4.57 ± 2.74 years.

During the follow-up period 177 (3.34%) participants experienced a major cardiovascular event (myocardial infarction, stroke, or cardiovascular death), including 44 (24.86%) cases of myocardial infarction, 110 (62.15%) cases of stroke, and 23 (12.99%) cases of cardiovascular death.

### 3.2. The Relationship Between Metabolic Syndrome Components and Major Cardiovascular Events

When assessing the number of metabolic syndrome components, individuals who met four criteria for metabolic syndrome were significantly more likely to experience a major cardiovascular event compared to those who met three criteria (*p* = 0.024) (Table 2).

Analysis of metabolic syndrome components revealed that individuals with elevated triglyceride level as one of the metabolic syndrome components were more likely to experience myocardial infarction, stroke, or cardiovascular death (*p* = 0.014). The presence of elevated glucose was also linked to an increased risk (*p* = 0.037) (Table 2).

Furthermore, when evaluating individuals diagnosed with metabolic syndrome based on different component combinations, those meeting the criteria for increased waist circumference, elevated triglyceride concentration, decreased HDL cholesterol concentration, and elevated blood pressure (WTHB) were significantly more likely to experience a major cardiovascular event (*p* = 0.038). Another statistically significant difference was observed for the WBG combination of MetS components: increased waist circumference, elevated blood pressure, and elevated glucose level (*p* < 0.001) (Table 2).

### 3.3. Effects of Specific Clusters of MetS Components on Arterial Stiffness

**Figure 1:**
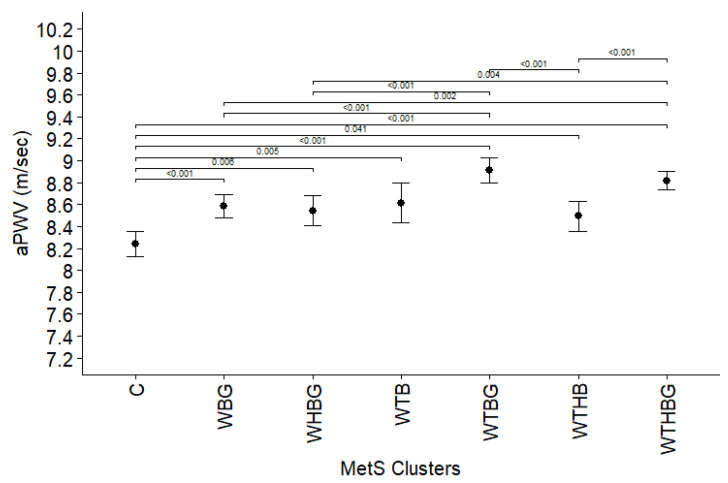
Arterial pulse wave velocity (represented as estimated marginal means and 95% CI) comparison between different metabolic syndrome component clusters, adjusted for age, gender, non-HDL cholesterol, smoking, and diabetes mellitus. C—group of low prevalence clusters (<5% population), W—increased waist circumference, T—elevated triglycerides, H—decreased high-density lipoprotein cholesterol level, B—high blood pressure, and G—elevated glucose level. Only *p* values < 0.05 displayed.

**Figure 2:**
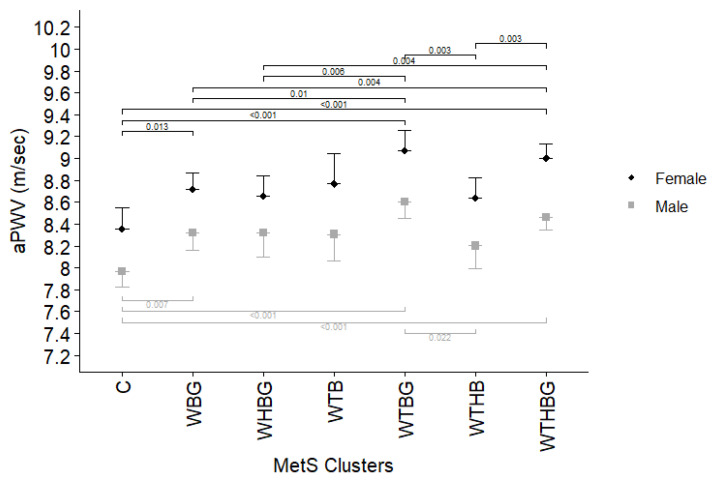
Arterial pulse wave velocity (represented as estimated marginal means and upper 95% CI for women/lower 95% CI for men) comparison between different metabolic syndrome component clusters for men and women separately, adjusted for age, gender, non-HDL cholesterol, smoking, and diabetes mellitus. C—group of low prevalence clusters (<5% population), W—increased waist circumference, T—elevated triglycerides, H—decreased high-density lipoprotein cholesterol level, B—high blood pressure, and G—elevated glucose level. Only *p* values < 0.05 displayed.

To examine the role that specific clusters of MetS components play for arterial stiffening, we also analyzed the association between the presence of various clusters and the indices of arterial stiffness. The comparison was adjusted for age, gender, non-HDL cholesterol, smoking, and diabetes mellitus. Analyzing aPWV and different metabolic syndrome component clusters for both genders (Figure 1), as well as for men and women separately (Figure 2), revealed that, for both genders, WBG, WTBG, and WTHBG clusters are related to higher aPWV value in comparison to low prevalence cluster group (prevalence < 5%), consisting of WTHG, THBG, WTH, WHB, THB, WTG, WHG, THG, TBG, and HBG (*p* < 0.05).

**Figure 3:**
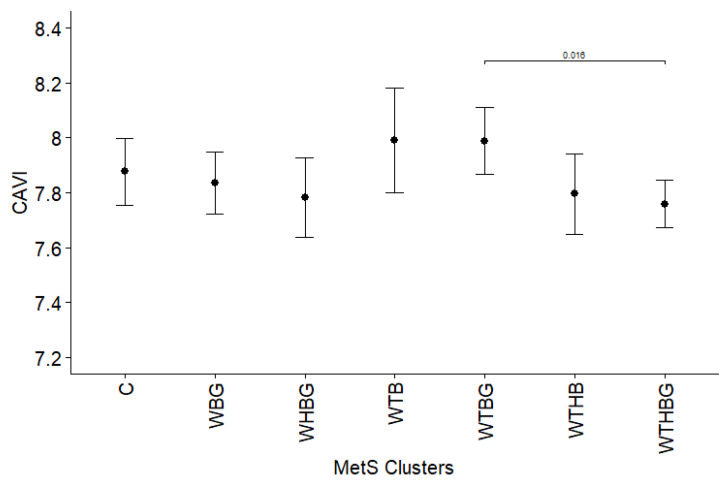
Cardio-ankle vascular index (represented as estimated marginal means and 95% CI) comparison between different metabolic syndrome component clusters, adjusted for age, gender, non-HDL cholesterol, smoking, and diabetes mellitus. C—group of low prevalence clusters (<5% population), W—increased waist circumference, T—elevated triglycerides, H—decreased high-density lipoprotein cholesterol level, B—high blood pressure, and G—elevated glucose level. Only *p* values < 0.05 displayed.

Performing a similar analysis with the aim of identifying relationships between CAVI and different metabolic syndrome component clusters showed a single statistically significant difference between WTBG and WTHBG. Subjects with WTBG had significantly higher CAVI value than those with WTHBG (7.99 vs. 7.76, *p* = 0.016) (Figure 3).

**Figure 4:**
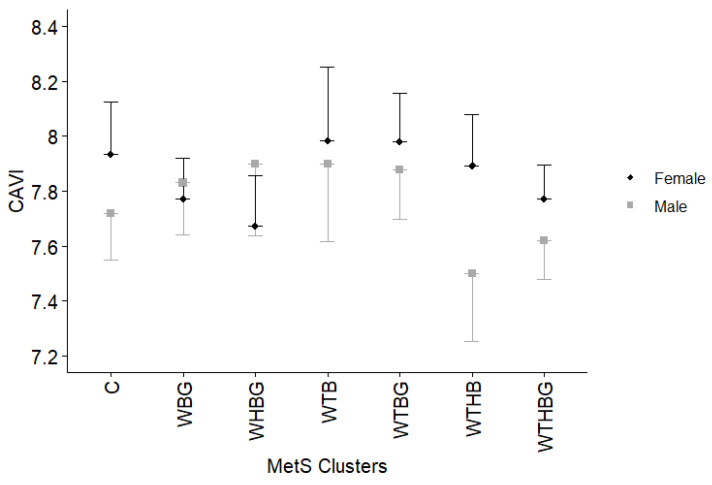
Cardio-ankle vascular index (represented as estimated marginal means and upper 95% CI for women/lower 95% CI for men) comparison between different metabolic syndrome component clusters for men and women separately, adjusted for age, gender, non-HDL cholesterol, smoking, and diabetes mellitus. C—group of low prevalence clusters (<5% population), W—increased waist circumference, T—elevated triglycerides, H—decreased high-density lipoprotein cholesterol level, B—high blood pressure, and G—elevated glucose level. No *p* values < 0.05 were found.

Analyzing relationships between CAVI and metabolic syndrome component combinations among men and women separately revealed no statistically significant differences (*p* > 0.05) (Figure 4).

### 3.4. The Relationship Between Metabolic Syndrome Components and Extremely Stiff Arteries

To evaluate the relationship between various metabolic syndrome component combinations and extremely stiff arteries, defined as aPWV above the 95th percentile (>11.3 m/s), logistic regression model was utilized with age, gender, non-HDL cholesterol, smoking, and diabetes mellitus used as confounding variables. Various metabolic syndrome component clusters were compared against a group of low prevalence clusters (prevalence <5%). This analysis showed that subjects with WTBG (OR = 2.351, 95% CI: 1.346–4.293, *p* = 0.004) and WTHBG (OR = 2.201, 95% CI: 1.334–3.854, *p* = 0.003) had more than twice higher odds for having extremely stiff arteries in comparison to low prevalence cluster group (Table 3). Subjects with extremely high aPWV also were older (56.25 ± 5.701 vs. 52.73 ± 6.452, *p* < 0.001) and had higher prevalence of diabetes mellitus (33.46% vs. 18.00%, *p* < 0.001), albeit these factors increased their odds of having extremely high aPWV less than two times.

Similar analysis was performed to evaluate the relationship between various metabolic syndrome component combinations and extremely stiff arteries, defined as CAVI higher than the 95th percentile (>10.2). We demonstrated that subjects with WTB (OR = 2.096; 95% CI: 1.120–3.846; *p* = 0.018) and WTBG (OR = 1.673; 95% CI: 1.037–2.750; *p* = 0.038) had statistically significantly higher odds for having extremely high CAVI in comparison to low prevalence cluster group. All confounding risk factors were also significant predictors of extremely high CAVI, with male sex standing out, as it increased the odds of CAVI >10.2 more than fivefold (Table 4).

## 4. Discussion

Cardiovascular events, including myocardial infarction, stroke, and cardiovascular-related mortality, continue to constitute the leading causes of morbidity and mortality on a global scale. Recent research [19] underscores the critical importance of arterial stiffness, measured through indices such as pulse wave velocity and the cardio-ankle vascular index, as a robust predictor of cardiovascular outcomes. These findings reinforce the concept that arterial stiffness serves as a pivotal determinant of cardiovascular risk [20]. The present study aimed at evaluating how these arterial stiffness parameters are linked to both increased CV risk and the presence of various MetS clusters.

Based on the findings of the present study, individuals meeting four criteria for metabolic syndrome exhibited a significantly higher risk of experiencing major cardiovascular events as compared to those meeting only three criteria (*p* = 0.024). These findings are consistent with previously published data, which demonstrate that an increasing number of MetS components correlate with a markedly elevated risk of cardiovascular complications [21]. This association may be attributed to systemic inflammation and insulin resistance, both of which intensify as the number of MetS components increases. Moreover, the results indicated that elevated triglyceride levels, one of the MetS components, were significantly linked to an increased risk of myocardial infarction, stroke, or cardiovascular death (*p* = 0.014). This observation is in line with recent evidence indicating that hypertriglyceridemia serves as a strong predictor of atherosclerotic events, even in individuals with normal cholesterol levels [22]. Additionally, a statistically significant difference was also observed for increased glucose as a MetS component (*p* = 0.037), which is consistent with prior studies showing that hyperglycemia significantly contributes to endothelial dysfunction and increased arterial stiffness [23].

Analysis of arterial markers and metabolic syndrome components demonstrated associations between cardiovascular risk surrogates and multiple MetS clusters. Nevertheless, only the WTHB and WBG clusters demonstrated statistically significant correlations with the occurrence of major cardiovascular events.

The WTHB cluster, characterized by central obesity, dyslipidemia, and hypertension, reflects core metabolic abnormalities driving atherogenesis and cardiovascular risk. These findings align with recent evidence emphasizing the compounded cardiovascular risk associated with concurrent metabolic disturbances, particularly when central obesity coexists with both hypertension and dyslipidemia [24]. Moreover, the strong contribution of central adiposity in high-risk clusters such as WTHB may reflect the long-term vascular consequences of early-life obesity, which initiates persistent metabolic and endothelial alterations, as reviewed by Carullo et al. [25]. These findings are further supported by Irace et al. [26], who demonstrated that BMI per se is not independently associated with carotid atherosclerosis after adjusting for metabolic syndrome components, emphasizing that fat distribution and metabolic profile, rather than overall weight, are more relevant predictors of vascular disease risk.

The WBG cluster’s significance highlights hyperglycemia as a key driver in metabolic syndrome pathophysiology. Chronic hyperglycemia fosters advanced glycation end-product formation, increasing endothelial permeability, suppressing nitric oxide synthase activity, and damaging cellular proteins [27,28]. The combined effects of these mechanisms impair vascular relaxation, thereby promoting the development of atherosclerosis [29,30].

When analyzing the association between various MetS clusters and arterial stiffness, we demonstrated that subjects with WBG, WTBG, and WTHBG clusters have higher aPWV values across both genders. Meanwhile, CAVI values showed no significant differences between most clusters including low prevalence combination group, except between the WTBG and WTHBG clusters (*p* = 0.016). This divergence in aPWV and CAVI findings likely reflects underlying methodological and physiological differences. Unlike aPWV, which is influenced by the arterial blood pressure at the time of measurement [31], CAVI has been designed to minimize dependence on current blood pressure [32]. Additionally, CAVI assesses stiffness along the arterial pathway from the aorta to the posterior tibial artery, reflecting both central elasticity and peripheral arterial tone [33]. Structural changes due to endothelial dysfunction and atherosclerosis primarily affect central arteries, while peripheral arteries are more susceptible to functional alterations over time [34]. This physiological distinction likely explains why aPWV, a marker more specific to central arterial stiffness, demonstrated greater sensitivity to metabolic disturbances in our cohort compared to CAVI.

Notably, most of the clusters associated with increased risk of CV event (WTHB and WBG) and with stiffer arteries in the cross-sectional analysis (WBG, WTBG, and WTHBG) are characterized by the presence of three principal components of metabolic syndrome: increased waist circumference, elevated blood pressure, and hyperglycemia, often accompanied by elevated triglycerides. Central obesity, indicated by a larger waist circumference, reflects increased visceral fat accumulation, which stimulates the secretion of pro-inflammatory cytokines such as interleukin-6 (IL-6) and tumor necrosis factor-alpha (TNF-α), leading to endothelial dysfunction and reduced arterial elasticity [35]. Simultaneously, both hypertension and hyperglycemia further contribute to arterial stiffening through oxidative stress and inflammatory pathways [36,37,38]. The interplay of these pathophysiological mechanisms provides a plausible explanation for why these clusters were shown to be among the least favorable for CV outcome or its surrogate marker, increased arterial stiffness. Furthermore, this is consistent with the further result of our study: as demonstrated by the logistic analysis, having WTBG, WTHBG, and WTB clusters more than twice increases the risk of extremely stiff arteries, also foregrounding the role of hypertension (B) on arterial stiffening.

Our study highlights that aPWV is a particularly sensitive marker of arterial stiffness in the context of metabolic syndrome. This observation is supported by widely accepted clinical guidelines: the 2024 European Society of Cardiology (ESC) and European Society of Hypertension (ESH) guidelines recommend the measurement of pulse wave velocity as a means of refining cardiovascular risk stratification and informing therapeutic decision-making [39]. In contrast, CAVI appears to reflect more chronic structural changes in the arterial wall. One possible explanation for the weaker associations observed between MetS clusters and CAVI in our study is that many participants may have been in relatively early stages of cardiovascular disease, where functional changes in arterial tone and compliance (captured by aPWV) precede structural alterations. Additionally, CAVI is less influenced by transient changes in blood pressure and more stable over time, which may reduce its sensitivity to the short- to medium-term metabolic disturbances characteristic of early MetS. This suggests that while CAVI remains valuable as a marker of long-term arterial health, aPWV may be more responsive to the initial vascular impact of MetS components, such as elevated blood pressure, glucose, and triglycerides. In summary, our findings suggest that aPWV is a more sensitive marker for detecting early arterial stiffness changes induced by MetS, whereas CAVI may require a longer duration of exposure to cardiovascular risk factors to reveal significant alterations.

The study by Scuteri et al. [11] also investigated the association between metabolic syndrome clusters and arterial stiffness markers. Our findings are largely consistent with their results, demonstrating that specific MetS combinations, namely WBG, WTBG, and WTHB, were associated with higher aPWV values compared to rare MetS patterns observed in less than 5% of cases. However, unlike Scuteri et al., who reported no significant differences when comparing these clusters to the full five-component MetS combination, our analysis identified significant differences between the WBG, WHBG, and WTHB clusters and the complete MetS cluster. When examining the male subgroup, our results further confirmed Scuteri et al.’s observation that specific clusters, namely WTBG and WTHBG, are associated with greater arterial stiffness compared to other, less frequent MetS combinations. Consistent with Scuteri’s findings, no significant difference was observed when comparing these clusters to the full five-component MetS cluster in men. Among women, our findings similarly mirrored the trend observed by Scuteri, with WBG, WTBG, and WTHBG clusters showing higher arterial stiffness relative to control group. Logistic regression analysis further revealed that the WTBG cluster (increased waist circumference, triglycerides, blood pressure, and glucose) and the WTHBG cluster (involving all five MetS components) were significantly associated with extremely elevated arterial stiffness. In contrast to Scuteri et al., who suggested that certain partial MetS combinations (e.g., TBW, GBW) may be stronger predictors of arterial stiffness than the full combination, our findings indicate a progressive increase in risk with the accumulation of MetS components. On top of that, our study provides several unique contributions to the existing literature. First, we assessed longitudinal outcomes, enabling evaluation of the relationship between MetS clusters and future risk of extremely elevated arterial stiffness. Second, we examined two complementary arterial stiffness indices, aPWV and CAVI, to provide a more comprehensive assessment of vascular health. Lastly, our study is based on a Lithuanian cohort, contributing valuable regional data from an underrepresented population in cardiovascular research. These novel aspects strengthen the generalizability and clinical relevance of our findings and highlight the need for tailored prevention strategies based on specific MetS patterns.

## 5. Conclusions

This study highlights arterial stiffness, particularly aPWV, as a sensitive early marker of cardiovascular risk in individuals with metabolic syndrome. Our findings demonstrate that increased arterial stiffness, particularly as measured by aPWV, correlates strongly with both the number and specific combination of MetS components; most notably, elevated triglycerides, glucose, and waist circumference, underscoring the cumulative vascular burden of hypertriglyceridemia, hyperglycemia, and central obesity. Moreover, subjects exhibiting MetS clusters WTBG, WTHBG, and WTB were more than twice as likely to have extremely stiff arteries. While aPWV effectively detected early vascular alterations, CAVI appeared less responsive to early metabolic changes and more indicative of advanced structural remodeling, suggesting it may reflect longer-term exposure to hemodynamic and metabolic stressors. These results support the use of pulse wave velocity in early risk stratification and highlight the urgency of early intervention to prevent progression to advanced vascular damage.

## Figures and Tables

**Table 1:** Metabolic syndrome component cluster distribution among study participants.

		Metabolic Syndrome Components				
		W	T	H	B	G
**Metabolic syndrome clusters**	**WTHBG** (*n* = 1657, 31.2%)	+	+	+	+	+
	**WTHB** (*n* = 519, 9.8%)	+	+	+	+	−
	**WTHG** (*n* = 81, 1.5%)	+	+	+	−	+
	**WTBG** (*n* = 710, 13.4%)	+	+	−	+	+
	**WHBG** (*n* = 497, 9.4%)	+	−	+	+	+
	**THBG** (*n* = 176, 3.3%)	−	+	+	+	+
	**WTH** (*n* = 44, 0.8%)	+	+	+	−	−
	**WTB** (*n* = 269, 5.1%)	+	+	−	+	−
	**WHB** (*n* = 211, 4.0%)	+	−	+	+	−
	**THB** (*n* = 55, 1.0%)	−	+	+	+	−
	**WTG** (*n* = 20, 0.4%)	+	+	−	−	+
	**WHG** (*n* = 26, 0.5%)	+	−	+	−	+
	**THG** (*n* = 20, 0.4%)	−	+	+	−	+
	**WBG** (*n* = 905, 17.0%)	+	−	−	+	+
	**TBG** (*n* = 81, 1.5%)	−	+	−	+	+
	**HBG** (*n* = 36, 0.7%)	−	−	+	+	+

W—increased waist circumference, T—elevated triglycerides, H—decreased high-density lipoprotein cholesterol level, B—high blood pressure, and G—elevated glucose level, +—metabolic syndrome component included in the cluster, −—metabolic syndrome component not included in the cluster.

**Table 2:** Metabolic syndrome components, their clusters, and their distribution between groups of subjects with and without major cardiovascular events.

Characteristic	Total (*n* = 5307)	Event (+) (*n* = 177)	Event (−) (*n* = 5130)	*p*
MetS (waist circumference), *n* (%)				0.764
yes	4939 (93.1)	164 (92.7)	4775 (93.1)	
no	368 (6.9)	13 (7.3)	355 (6.9)	
MetS (triglycerides), *n* (%)				**0.014**
yes	3632 (68.4)	136 (76.8)	3496 (68.1)	
no	1675 (31.6)	41 (23.2)	1634 (31.9)	
MetS (HDL-C), *n* (%)				0.155
yes	3322 (62.6)	120 (67.8)	3202 (62.4)	
no	1985 (37.4)	57 (32.2)	1928 (37.6)	
MetS (blood pressure), *n* (%)				0.415
yes	5116 (96.4)	173 (97.7)	4943 (96.4)	
no	191 (3.6)	4 (2.3)	187 (3.6)	
MetS (glucose), *n* (%)				**0.037**
yes	4209 (79.3)	129 (72.9)	4080 (79.5)	
no	1098 (20.7)	48 (27.1)	1050 (20.5)	
MetS (number of components), *n* (%)				**0.024**
5	1657 (31.2)	55 (31.1)	1602 (31.2)	
4	1983 (37.4)	81 (45.8)	1902 (37.1)	
3	1667 (31.4)	41 (23.2)	1626 (31.7)	
MetS (WTHBG), *n* (%)				1.000
yes	1657 (31.2)	55 (31.1)	1602 (31.2)	
no	3650 (68.8)	122 (68.9)	3528 (68.8)	
MetS (WTHB), *n* (%)				**0.038**
yes	519 (9.8)	26 (14.7)	493 (9.6)	
no	4788 (90.2)	151 (85.3)	4637 (90.4)	
MetS (WTHG), *n* (%)				1.000
yes	81 (1.5)	2 (1.1)	79 (1.5)	
no	5226 (98.5)	175 (98.9)	5051 (98.5)	
MetS (WTBG), *n* (%)				0.177
yes	710 (13.4)	30 (16.9)	680 (13.3)	
no	4597 (86.6)	147 (83.1)	4450 (86.7)	
MetS (WHBG), *n* (%)				0.600
yes	497 (9.4)	14 (7.9)	483 (9.4)	
no	4810 (90.6)	163 (92.1)	4647 (90.6)	
MetS (THBG), *n* (%)				0.194
yes	176 (3.3)	9 (5.1)	167 (3.3)	
no	5131 (96.7)	168 (94.9)	4963 (96.7)	
MetS (WTH), *n* (%)				0.404
yes	44 (0.8)	0 (0.0)	44 (0.9)	
no	5263 (99.2)	177 (100.0)	5086 (99.1)	
MetS (WTB), *n* (%)				1.000
yes	269 (5.1)	9 (5.1)	260 (5.1)	
no	5038 (94.9)	168 (94.9)	4870 (94.9)	
MetS (WHB), *n* (%)				0.073
yes	211 (4.0)	12 (6.8)	199 (3.9)	
no	5096 (96.0)	165 (93.2)	4931 (96.1)	
MetS (THB), *n* (%)				1.000
yes	55 (1.0)	1 (0.6)	54 (1.1)	
no	5252 (99.0)	176 (99.4)	5076 (98.9)	
MetS (WTG), *n* (%)				0.142
yes	20 (0.4)	2 (1.1)	18 (0.4)	
no	5287 (99.6)	175 (98.9)	5112 (99.6)	
MetS (WHG), *n* (%)				1.000
yes	26 (0.5)	0 (0.0)	26 (0.5)	
no	5281 (99.5)	177 (100.0)	5104 (99.5)	
MetS (THG), *n* (%)				1.000
yes	20 (0.4)	0 (0.0)	20 (0.4)	
no	5287 (99.6)	177 (100.0)	5110 (99.6)	
MetS (WBG), *n* (%)				**<0.001**
yes	905 (17.1)	14 (7.9)	891 (17.4)	
no	4402 (82.9)	163 (92.1)	4239 (82.6)	
MetS (TBG), *n* (%)				1.000
yes	81 (1.5)	2 (1.1)	79 (1.5)	
no	5226 (98.5)	175 (98.9)	5051 (98.5)	
MetS (HBG), *n* (%)				1.000
yes	36 (0.7)	1 (0.6)	35 (0.7)	
no	5271 (99.3)	176 (99.4)	5095 (99.3)	

HDL-C—high-density lipoprotein cholesterol. W—increased waist circumference, T—elevated triglycerides, H—decreased high-density lipoprotein cholesterol level, B—high blood pressure, and G—elevated glucose level.

**Table 3:** Specific clusters of metabolic syndrome components as determinants of extremely stiff arteries (aPWV > 11.3 m/s), adjusted for age, gender, non-HDL cholesterol, smoking, and diabetes mellitus.

	Prevalence in Population	Prevalence in Extremely Stiff Arteries Group	OR	95% CI	*p*
Age			1.086	1.054–1.122	**<0.001**
Male Gender			1.010	0.661–1.539	0.965
Smoking			0.757	0.527–1.064	0.119
Non-HDL-C			1.047	0.948–1.539	0.360
Diabetes Mellitus			1.795	1.353–2.368	**<0.001**
WBG	17.1	18.3	1.671	0.962–3.038	0.078
WHBG	9.4	7.6	1.213	0.620–2.395	0.573
WTB	5.1	3.0	1.283	0.514–2.947	0.570
WTBG	13.4	17.5	2.351	1.346–4.293	**0.004**
WTHB	9.8	4.6	0.884	0.406–1.867	0.749
WTHBG	31.2	42.6	2.201	1.334–3.854	**0.003**

HDL-C—high-density lipoprotein cholesterol. W—increased waist circumference, T—elevated triglycerides, H—decreased high-density lipoprotein cholesterol level, B—high blood pressure, and G—elevated glucose level.

**Table 4:** Specific clusters of metabolic syndrome components as determinants of extremely stiff arteries (CAVI > 10.2), adjusted for age, gender, non-HDL cholesterol, smoking, and diabetes mellitus.

	Prevalence in Population	Prevalence in Extremely Stiff Arteries Group	OR	95% CI	*p*
Age			1.137	1.100–1.176	**<0.001**
Male Sex			5.083	3.340–7.793	**<0.001**
Smoking			1.586	1.187–2.108	**0.002**
Non-HDL-C			1.108	1.001–1.222	**0.044**
Diabetes Mellitus			1.694	1.242–2.289	**<0.001**
WBG	17.1	15.3	1.125	0.677–1.890	0.652
WHBG	9.4	10.5	1.483	0.839–2.612	0.172
WTB	5.1	7.7	2.096	1.120–3.846	**0.018**
WTBG	13.4	20.2	1.673	1.037–2.750	**0.038**
WTHB	9.8	6.0	0.898	0.459–1.693	0.744
WTHBG	31.2	29.0	1.072	0.686–1.717	0.766

HDL-C—high-density lipoprotein cholesterol. W—increased waist circumference, T—elevated triglycerides, H—decreased high-density lipoprotein cholesterol level, B—high blood pressure, and G—elevated glucose level.

## Data Availability

The data underlying this article cannot be shared publicly due to the privacy of individuals that participated in the study. The data will be shared on reasonable request to the corresponding author.
